# Technology‐driven 5G enabled e‐healthcare system during COVID‐19 pandemic

**DOI:** 10.1049/cmu2.12240

**Published:** 2021-06-05

**Authors:** Nasser Alshammari, Md Nazirul Islam Sarker, M.M. Kamruzzaman, Madallah Alruwaili, Saad Awadh Alanazi, Md Lamiur Raihan, Salman Ali AlQahtani

**Affiliations:** ^1^ Department of Computer Science, College of Computer and Information Sciences Jouf University Sakakah Saudi Arabia; ^2^ School of Political Science and Public Administration Neijiang Normal University Neijiang China; ^3^ Department of Computer Engineering and Networks, College of Computer and Information Sciences Jouf University Sakakah Saudi Arabia; ^4^ Laboratory of Sustainable Rural Development, Graduate School of Global Environmental Studies Kyoto University Kyoto Japan; ^5^ College of Computer and Information Sciences King Saud University Saudi Arabia

## Abstract

Technology‐driven control measures could be an important tool to control the COVID‐19 pandemic crisis. This study evaluates the potentiality of emerging technologies such as 5G and 6G communication, Deep Learning (DL), big data, Internet of Things (IoT) etc. for controlling the COVID‐19 transmission and ensuring health safety. The healthcare sector is able to provide a unified, rapid, and incessant service to people by applying modern wireless connectivity tools like 5G or 6G during the COVID‐19 pandemic. This study has identified eight key areas of applications for the COVID‐19 management like infection detection; travel history analysis; identification of infection symptoms; early detection; transmission identification; access to information in lockdown; movement of people; and development of medical treatments and vaccines. Data have been collected from the respondents living in Sakaka city, KSA during pandemic. This study reveals that most people receive information from social networking sites, health professionals, and television without facing any challenges. The analysis shows that, during the COVID‐19 pandemic, about 42% of respondents felt tense always or most of the time in a day. Only 28.6% of respondents felt tense sometimes, whereas the remainder (about 30%) did not feel tense in relation to the COVID‐19 crisis. Satisfaction with COVID‐19‐related information is also positively correlated with COVID‐19‐related information literacy (*r* = 0.53, *p* < 0.01) that is also positively correlated with depression or emotion, anxiety, and stress (*r* = ‐0.15, *p* < 0.05). The long‐term pandemic is creating several psychological symptoms including anxiety, stress, and depression, irrespective of age.

## 1 INTRODUCTION

The entire world is now facing a terrible and fatal COVID‐19 pandemic caused by SARS‐CoV‐2. From its discovery in Wuhan, China, in December 2019, COVID‐19, as a rapidly transmitted infectious coronavirus, has created panic worldwide. As on May 5, 2021, the total number of identified cases is 154,988,353 and death is 3,241,164 [1]. At the current time, the number of cases is increasing rapidly. Traditional technologies in the medical field have failed to control this pandemic. Scholars, think‐tanks, policy makers, and practitioners are searching for new technologies to control the attack of this coronavirus. At this moment, timely accurate information and measures are necessary for practitioners and healthcare settings as they seek ways for people to assist in managing this pandemic. Advanced digital technology could be a key tool to stop the rapid transmission, to control COVID‐19, to provide treatment, and to develop medicines and vaccines. Access to information could also make people more aware of steps needed for preparation for, protection against, and prevention of COVID‐19. Only the available advanced technologies can ensure people's access to COVID‐19‐related information. According to many scholars [2, 3, 4, 5, 6, 7, 8], COVID‐19‐related information should be made available to people through the use of advanced digital technologies, comprising big data, artificial intelligence (AI), Deep Learning (DL), Internet of Things (IoT), 5G and 6G. These technologies are necessary to solve the main clinical problems as well as removing barriers to people's access to information.

With the COVID‐19 pandemic given the utmost priority, scholars and practitioners are working hard in their attempts to develop effective measures and strategies to address the life‐threatening coronavirus worldwide. Advanced digital technology could help to develop modern diagnostic appliances and treatments. The effective use of advanced digital technology could also have a rapid effect on the control of this global pandemic. The process of developing context‐specific technology should be continued to improve outcomes. Digital technology not only helps to address the COVID‐19 pandemic but is also important for the social and economic dimensions of people's lives during the pandemic [9, 10]. This pandemic is showing the weaknesses of health sectors, irrespective of whether these sectors are in developed or developing countries. Governments of many countries have given top priority to the development of advanced technology to cope with this crisis. Only a few have achieved a small amount of success, with most having failed to control the coronavirus's rapid transmission, despite applying social distancing, screening, mass testing, contact tracing, and lockdowns. In one example, South Korea used digital technologies to address the COVID‐19 pandemic particularly for surveillance, testing, screening, and quarantine, and achieved a fruitful outcome.

Digital technology (DT) is a crucial part of daily life. Rapid DT developments have created demand for every sector. Deep Learning is a part of Artificial Intelligence (AI) in broader aspect [11, 12]. Symbolic Structures are included in the scope of AI along with DP. Symbolic Systems use symbol manipulation and logic to create intelligent actions. Expert Structures and Information Graphs are examples. Expert Systems make decisions based on if‐else rules. In graph data structures, Information Graphs store relationships between objects. The use of Knowledge Graphs of Benevolent AI is tremendously beneficial for combating COVID‐19. Health information technology (HIT) covers a number of related fields like information, health and emerging technologies, all of which are experiencing technological transformation [2]. In reality, this transition is a move away from conventional medical practices and toward the use of new technology to improve growth at all stages of the healthcare process. HIT encompasses a variety of cutting‐edge technology, including e‐health, m‐health, telemedicine, social networking sessions, email, and texting. These innovations offer people seeking medication and health care a huge advantage [13].

Information systems (ISs) are playing a vital role in the COVID‐19 pandemic, more so than any other system in the healthcare industry. These systems help to manage data to convey messages as rapidly as required. They also ensure that evidence is available for making decisions, formulating policy and strategy, implementing action, and coordinating other elements to strengthen healthcare through intelligence [14]. In order to enable smart healthcare applications, the existing healthcare system depends largely on 5G (fifth‐generation) networks; though it cannot backup the fast development of the related applications for long time due to limited bandwidth resources. In relations to bandwidth, efficiency, expectancy, and rate of data, sixth generation (6G) networks deliver improved services compared to 5G [15]. 5G facilities are generally classified into the eMBB (enhanced mobile broadband), the URLLC (ultra‐reliable low latency) and the massive communications of the machine type (mMTC) [16]. It can provide an internet connectivity for medical devices, video calling for ensuring telemedicine through mMTC. It also supports to surveillance for infectious diseases like COVID‐19 through various autonomous and drone vehicles. Nonetheless, the services provided by 5G are inadequate to meet both the development of healthcare skills and disaster circumstances. In particular, remote healthcare privacy and security, ubiquitous telemedicine communication in areas with inadequate infrastructure, Internet of Medical Things (IoMT) device connectivity, and very reliable and little expectancy communication for remote surgical treatment with mixed reality [17, 18].

Digital technology and automation can provide better health facilities particularly in this pandemic [19]. People can only receive better services if the authorities can establish advanced information systems (ISs). When caring for vulnerable people, an information system (IS) can ensure access for these people, as well as responsiveness and health‐related suggestions through providing accurate, rapid, fruitful, coordinated, and real‐time data. Health inequality in a community or a state can be removed only by ensuring that people have easy access to an information system (IS). Information technology (IT) is based on data, so the governance of IT requires regular data production, collection, management, and analysis. Simultaneously, infrastructure should be established through the adoption of advanced technology, computer applications, and a real‐time database. This process requires the integration and adjustment of modern technologies, such as big data, AI, deep learning, IoT, and machine learning. The current study considers all the available technologies that are embedded within an information system (IS). People, society, and the state are dependent on the entire IS for gaining easy access to information related to COVID‐19 and emergency treatment.

Many published documents have already been focused on the epidemiology of coronavirus infection for networking demand [20], an expert's opinion on coronavirus [21], information about infectious diseases [22], and online information seeking [22, 23], patient monitoring, but the focus on healthcare communication technology and information access are still lacking worldwide. As almost all cities in KSA have already faced coronavirus infection, it is high time for the availability and reliability of information, and people's satisfaction with that information to be assessed to help them obtain beneficial information for their protection from COVID‐19 infection. This study contributes to the policy level by providing context specific recommendations of information access with empirical evidence from those at the centre of technological development. Therefore, as previously stated, this study intends to evaluate the potentiality of emerging digital technologies for controlling COVID‐19 transmission and ensuring health safety.

## 2 RELATED WORKS

### 2.1 5G communication system for controlling COVID‐19

5G (Fifth‐generation) wireless communication facilities and the related application situations are currently under debate [17]. One of the key motivations is to advance 5G communication and IoT from a social and humanitarian point of view, that is, to grow communication system to develop the life quality of people. It can help to improve the smart healthcare system's strong communication infrastructure in relations of better reliability, stability of connections, ultra‐massive accessibility, scalability of the network and rapid response flexibility, enabling monitoring and preventive action to be taken in pandemic situations [18].

According to Wijesooriya et al. [24], telehealth can be an effective approach to ensure better health care and train the medical professionals at the time of pandemic. Rahman et al. [25] also recommend to use deep learning approach for ensuring better health services though the latest technology under 5G. The wireless communication technologies can help to monitor the spread of viruses, to improve the health, treatment process and socio‐economic sectors. In disaster scenarios, the huge number of treatment seeker visited to the hospital quickly overwhelms hospitals. It is difficult to control the situation because of the scarcity of medical personnel and equipment along with a minimum patient capacity [17]. In pandemic cases, in fact, things get inferior because of the rapid transmitted capacity of virus. Minimizing the physical contact between treatment seeker and physicians is an effective way to avoid this problem. It can be achieved by using IoMT and robots in the hospital and diagnostic centres. These devices can also help to monitor and transfer health records of patients over the internet to physicians, and ensuing to less interactions. Cloud infrastructure can be utilized to many cases, like providing equipment, health checkups and sanitizing the region of the patient for possible monitoring [26].

### 2.2 Deep learning for COVID‐19 pandemic control

The healthcare sector is rising rapidly with the help of wireless and deep learning (DL) technology [27]. It's now a multibillion‐dollar company. Particularly in developing countries, where the low‐income people are growing, the industry is flourishing. The demand of treatment seekers is versatile and they always want to get easily accessible health care with a friendly environment. Due to modern life, people have very less time for routine checkups to consult a specialist healthcare professional. Massive traffic jams and a shortage of qualified doctors are major factors that discourage people from visiting hospitals. Sometimes treatment seekers need to visit an expert physician in another country that can easily manage by video chatting with the physician using smart technology. But some infectious diseases need to be handled with teamwork. For example, COVID‐19 must be handled by case‐wise. Treatment seekers, physicians, and stakeholders can be smartly linked for organized management. The healthcare sector is able to provide a unified, rapid, and incessant service to people by applying modern wireless connectivity tools like 4G or 5G. By offering various significant data about the treatment seekers and the community, the IoTs introduced a new way to the operation. There is now less storage capacity headache; however, one crucial problem exists, like, the bandwidth requires by the immense volume of treatment seeker's data.

### 2.3 Digital technology driven COVID‐19 pandemic control

The coronavirus (COVID‐19) is now a global pandemic owing to the nature of its transmission, and its infection and mortality rates. This pandemic has become the most serious health hazard for human beings since last century. Modern healthcare is based on information. Not only is information applicable for practitioners and scholars but also for members of the general public seeking healthcare. The healthcare industry depends mainly on information for the treatment process, diagnosis, disease prevention, and protection from disease, as well as being actively involved in and reliant on the acquisition, storage, analysis and interpretation of information. Jacobs et al. [28] conducted a study on healthcare‐seeking behaviour and concluded that people tend to collect information from the internet and traditional media. However, they found that the internet should not be used when seeking healthcare information owing to its poor level of authenticity and creation of inequality. Conversely, due to long‐term lockdowns and communication barriers, people would like to readily access information to fight against COVID‐19. At times, the adoption by people of non‐scientific information from the internet or social media has created major problems.

**FIGURE 1:**
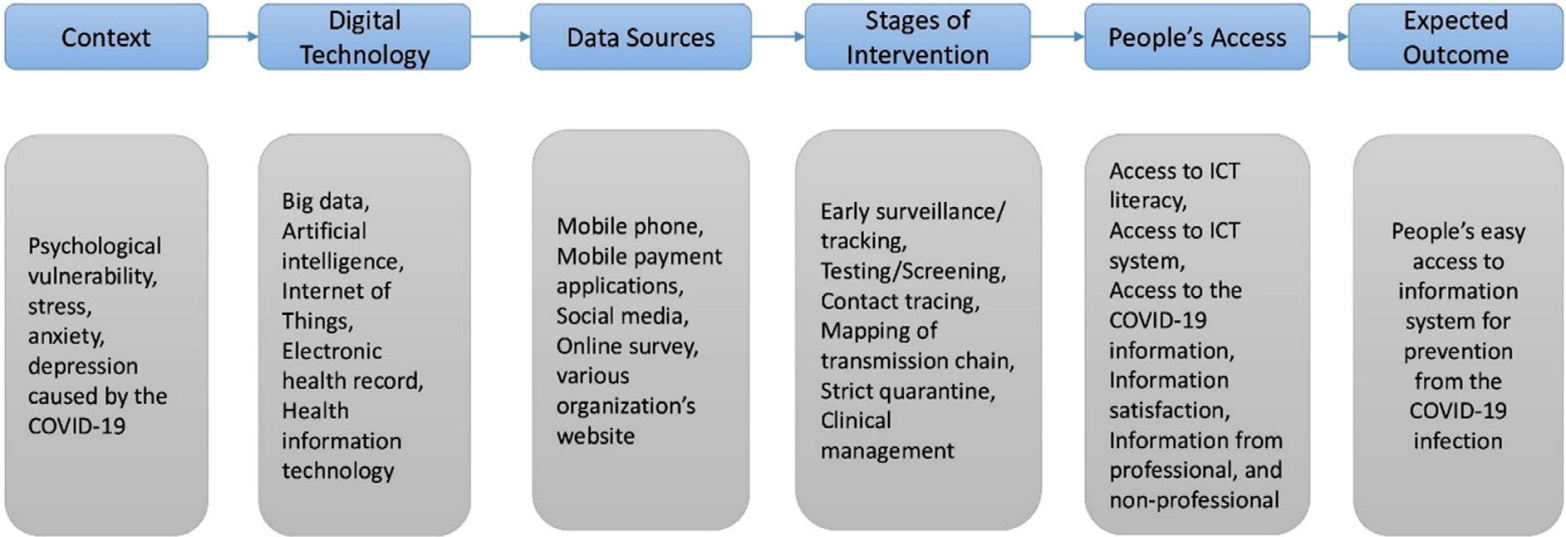
Major steps of technology‐driven 5G enabled COVID‐19 pandemic control

A health information system (IS) allows a practitioner to use and share electronic information about a patient with various information systems (ISs) [29, 30]. This brings several benefits, such as ease in keeping patient information for a long time, ease in handling huge amounts of data, the forecasting of disease severity, and easy diagnosis and treatment. However, the challenges include the absence of interoperability, and requirements for experts, digital devices, and a very large amount of investment. Tao et al. [31] reported several factors, such as self‐efficacy, subjectivity, beliefs, facilities, and adoption behaviour which directly affect people's acceptance of health technology. These authors argued that the place of origin, the user, and the type of technology also influence people's health information acceptance. Due to the rapid development of IT, information technology (IT) has created its own space in every sector including health and is transforming societal dependence from conventional practices to modern information communications and technology (ICT). The health sector is also using these benefits that have significantly increased during the COVID‐19 pandemic (Figure 1). Social media and the internet are flooded with various kinds of health‐related information which is easily available to people worldwide. However, the authenticity of much of this information is not verified, so people continue to need trusted information from health professionals [32]. Mobile health is the latest technological development in the healthcare industry through which the information seeker can obtain information from a health professional through a desktop computer or laptop. Electronic health (e‐health) and mobile health (m‐health) use the latest communications technology to ensure that health information seekers have easy access to health `information systems (ISs) and services [33]. Furthermore, Sebetci [34] conducted a study on users’ satisfaction with information systems (ISs) and argued that the major influencing factors were the quality of the information and the system, the resources available, and the technology being used. The development of these factors can ensure the improvement of users’ satisfaction with health information.

### 2.4 Regional covid‐19 management using digital technology

Abolhassani et al. [19] investigated older people's acceptance of health ICT, reporting that it was accepted by older educated people without any concerns. According to Hossain and Muahmmad [35], emerging digital technologies can help health‐related stakeholders to develop innovative solutions for health services. These technologies can ensure the development of information systems (ISs) for people's access as well as advanced technological supports through big data, AI, the IoT etc. Alsadan et al. [2] analysed the health information technology (HIT) available in Arab countries and reported poor adoption of HIT in the Arab healthcare industry, with public hospitals less professional in using digital technology than private hospitals. Using emerging technology for management of the COVID‐19 pandemic has already proved critical in a few advanced countries, such as South Korea, China, Taiwan, Japan, etc. Digital technology comprises big data, the IoT, the mathematical forecasting model, nano technology, and telemedicine, along with easy access to information systems (ISs). AI can be a key tool in tackling the COVID‐19 crisis through its assistance in rapid decision making and avoiding the rapid transmission of the coronavirus. It can also help in understanding the vaccine development process. However, according to Naude [14], AI cannot be fruitful in COVID‐19 crisis management due to the exaggerated amount of unnecessary data and the scarcity of appropriate data. Aceto et al. [13], in their study on Industry 4.0 and health, concluded that information technology (IT) could support information systems (ISs) through IoT, big data, artificial intelligence (AI), and cloud computing applications to ensure better healthcare. Traditional healthcare approaches are being transformed to technology‐based smart healthcare through the adoption of advanced digital technologies. Chen et al. [36] investigated how big data could help to improve healthcare services and the challenges they face. These authors reported that timely preventive services could be ensured through big data and how to set up the air quality‐aware application scenarios. An enormous amount of data has been produced in the COVID‐19 pandemic from health surveillance, which does not consider defensive models against adversarial perturbations [37]. According to Hua and Shaw [38], data‐driven digital technologies need to be combined well with strict governance and regulations, as well as with people's participation, to ensure people's smooth access to information systems (ISs), thus assisting in preventing future pandemics like COVID‐19.

### 2.5 Potential application of data‐driven digital technology

After analyzing the available digital technology through the extensive literature review, this study identified eight key areas of applications for COVID‐19 management. These comprised infection identifications; symptoms of infection; travel history; identification of transmission; early detection; people's movements; ready information in lockdown period; and development of treatments and vaccines [39]. These applications have been frequently used in the healthcare industry and are applicable to identifying, preventing, protecting against, and controlling the COVID‐19 pandemic, while ensuring information system access (Table 1).

**TABLE 1:** Potential emerging technologies for COVID‐19 pandemic management

Sl. No.	Applications	Description
1	Infection identification	Big data and artificial intelligence help to identify infected people and keep records for further use.
2	Symptoms of infection	Big data typically stores information on infection and associated symptoms of individuals, which can aid in identifying the infected person.
3	Travel history	Travel history aids in identifying and controlling coronavirus transmission by identifying the individual who comes into contact with the infected person.
4	Identification of transmission	Since COVID‐19 is a rapidly transmitted coronavirus, big data and artificial intelligence (AI) can aid in identifying and controlling the virus's transmission methods and areas.
5	Early detection	Big data aids in the monitoring of infection patterns and early detection.
6	Movement of people	Movements of people can be tracked easily with the aid of big data and AI technology, which can also assist in identifying areas where pathogens may be present.
7	Information availability in lockdown period	Due to the fact that most people were forced to stay at home for a lockdown, they required immediate information, which emerging technologies can provide easily.
8	Development of medical treatments and vaccines	Treatments and vaccines can be developed through using advanced digital technology.

## 3 METHODOLOGY

### 3.1 Geographical features

Sakaka city is situated in the Kingdom of Saudi Arabia (KSA) in the northern part of the Greater Nufud desert, covering nearly 100 km^2^. The city, located 980 km north of Riyadh and 1,286 km north of Jeddah, is 566 m (1,857 ft) above sea level [40]. The population of Sakaka city is about 280,000, comprising almost 57% of the total population of the Al Jouf region. The government of Saudi Arabia has invested an enormous amount to develop it as a smart city. Although Sakaka city covers nearly 100 km^2^, the construction area of the city is around 57 km^2^. Of this land, 58% comprises the area on which buildings are constructed, whereas 38% comprises open space and 4% is unoccupied land [40].

### 3.2 Population and sample

This study considers people living in Sakaka city, such as university teachers, graduate students, government employees, non‐government employees, municipal employees and personnel, the general public, and leaders of society as the study population. The sample size has been determined using the standard formula stated below:

$$n\;=\frac{z^{2}pq}{d^{2}}\;$$

$$n\;=\frac{{\left(1.96\right)}^{2}\times 0.5\times 0.5}{{\left(0.07\right)}^{2}}\;$$

n=196

where

*n* = the desired sample size
*z* = the standard normal deviate, set at 1.96
*p* = estimated to be at 50% level.
*q* = 1‐*p*
*d* = degree of accuracy, set at 7%

The estimated sample is 196; therefore, 196 respondents have been included in this study.

### 3.3 Measurement approaches

Several approaches and indicators have been selected based on the extensive literature, expert consultation, and extensive visits to the study area. Indicators of various measurements have been modified according to the context of the COVID‐19 pandemic.

#### 3.3.1 Demographic characteristics, selection, and measurement

This study identified a selection of demographic characteristics of citizens living in the Sakaka city. The selected demographic characteristics comprised: age, gender, education level, years lived in Sakaka city, profession, annual income, and organizational participation.

#### 3.3.2 People's access to covid‐19‐related information

People's access to information related to COVID‐19 was determined by employing six questions with a range of indicators. These comprised: main sources of information, seeking information from non‐health professionals, usefulness of information from non‐health professionals, types of information, overall access to information, and information barriers.

#### 3.3.3 COVID‐19 information satisfaction

Several statements about satisfaction with COVID‐19 information were included. These statements sought responses including respondents’ satisfaction with the amount of information, the quality of information and the available sources of information. A 5‐point Likert scale was applied to measure people's information satisfaction for each of these statements. The scores ranged from 0 to 5, with 5 indicating a higher level of satisfaction while a lower score showed a lower level of satisfaction.

#### 3.3.4 COVID‐19‐related literacy

Respondents’ level of literacy on COVID‐19 was measured based on six statements, comprising their level of understanding; sharing of COVID‐19‐related thoughts with others; credibility of COVID‐19‐related information; checking the authenticity of information; making decisions based on this information; and using this information in daily life. A 4‐point scale, ranging from ‘never’, ‘rarely’, and ‘sometimes’ to ‘often,’ rated respondents’ literacy level. A high score showed that the respondent had a higher level of COVID‐19 literacy while a low score showed a lower level of literacy.

### 3.4 COVID‐19‐related depression, anxiety, and stress

The study adopted the Depression Anxiety Stress Scales (DASS), developed by Lovibond and Lovibond, with appropriate modification in accordance with the research context [41]. A 4‐point scale was used to rate several depression indicators such as: always feeling tense; feeling a slow‐down in study; still the same enjoyment as before COVID‐19; always frightened; lost interest on study; laughing at funny things; feeling restless; always thinking in his/her mind; looking forward to enjoying study; feeling cheerful with study; sudden panic; relaxed during studying; and enjoying the radio and TV programs. A high score showed high levels of depression, anxiety, and stress among respondents about COVID‐19 while a low score showed a low level of depression, anxiety, and stress.

### 3.5 Data analysis

The latest version of SPSS (IBM SPSS Statistics) was used to analyse the collected data in order to present the results. Descriptive statistics were also done to present respondents’ demographic characteristics, access to COVID‐19‐related information, COVID‐19‐related literacy, satisfaction with the information, and DASS scores. Values were expressed as percentages, means, and standard deviations. Pearson's correlation coefficients were applied to show the relationships between different variables.

### 3.6 Ethical approval

A formal ethical clearance was obtained from the Ethics Committee of Jouf University, Kingdom of Saudi Arabia (KSA).

## 4 RESULTS OF THE STUDY

### 4.1 Demographic characteristics of respondents

The respondents’ selected key demographic characteristics related to digital technology adoption during the COVID‐19 pandemic for information access and protection from possible infection. Most respondents (87.8%) were male and middle‐aged (aged 31–50 years). Most of them (59.2%) had Bachelor degrees and were married (84.2%). Most (91.8%) had already spent 3–5 years in Sakaka city, Kingdom of Saudi Arabia (KSA). Most respondents (56.1%) had a large family size with more than four members. About 74% of respondents had no health insurance, with only 26% having health insurance. All respondents had smartphones and internet connections and most (80%) had laptops, while 35% had a computer and 36% had a tablet (Table 2).

**TABLE 2:** Demographic features of respondents

Demographic characteristics	Categories	No.	Percentage	Mean	Standard deviation
Gender	Male	172	87.8	1.12	0.33
	Female	24	12.2		
Age	Young (18–30)	44	22.4	36.30	8.64
	Middle‐aged (31–50)	141	71.9		
	Older (above 50)	11	5.6		
Education level	Secondary	16	8.2	15.64	2.44
	Diploma	20	10.2		
	Bachelor	116	59.2		
	Master's	35	17.9		
	Doctorate	9	4.6		
Health insurance	Yes	50	25.5	1.26	0.44
	No	146	74.5		
Duration of stay in Sakaka city	Short duration (0–2 years)	16	8.2	3.73	0.75
	Medium duration (3–5 years)	180	91.8		
	Long duration (above 5 years)	0	0		
Family size	Single (1)	9	4.6	5.34	2.93
	Couple (2)	9	4.6		
	Medium family (3–4)	68	34.7		
	Big family (above 4)	110	56.1		
Marital status	Yes	165	84.2	1.84	0.37
	No	31	15.8		
Access to ICT	Computer	69	35.2	1.35	0.48
	Laptop	157	80.1	1.80	0.40
	Smartphone	196	100	2.0	0
	Tablet	70	35.7	1.35	0.48
	Internet connection	196	100	2.0	0

Source: Field survey, 2020.

### 4.2 COVID‐19‐related information sources, and access

Access to information on the COVID‐19 pandemic was assessed in terms of the main sources of information, information‐seeking behaviours, information usefulness and types, and overall access as well as challenges faced in obtaining information (Table 3). Multiple responses were considered to identify the main sources of information. The major prelisted sources were television (TV), radio, newspapers, hospital, mosque, community meetings, and social networking. Most respondents (96%) used social networking sites for COVID‐19‐related information, along with radio (83%) and television (75%). About 43% of respondents obtained information from a school or workplace, while the information source for 40% was the mosque and, for 60%, community meetings. Hospital was thought to be a trusted place for pandemic‐related information as doctors and nurses worked there. However, only 20% of respondents sought pandemic information from a hospital, while 26% of respondents received it from their daily newspaper. It was also found that about 66% of respondents sought information from health professionals, with only 5% arguing for the usefulness of information from non‐health professionals.

**TABLE 3:** Access to COVID‐19‐related information

Variables	Major indicators	Response	Frequency	Percentage
Main sources of information	Television	Yes	147	75
	Radio	Yes	162	82.7
	Newspapers	Yes	51	26
	Hospital (doctor or nurse)	Yes	39	19.9
	School	Yes	85	43.4
	Church/mosque	Yes	78	39.8
	Community meetings	Yes	117	59.7
	Social networking	Yes	188	95.9
Seeking information	Obtained from non‐health professional	Yes	9	4.6
		No	130	66.3
		Maybe	57	29.1
Usefulness of information	Obtained from non‐health professional	Yes	18	9.2
		No	103	52.6
		Maybe	75	38.3
Types of information	Method of infection	Yes	146	74.5
	Symptoms of COVID‐19 infection	Yes	12	6.1
	Medicine for COVID‐19 treatment	Yes	53	27
	Regular health exercise	Yes	35	17.9
	Personal hygiene	Yes	80	40.8
	Home treatment	Yes	80	40.8
	Isolation	Yes	109	55.6
	Social distancing	Yes	139	70.9
Overall access to information	No		5	2.6
	Poor		6	3.1
	Good		41	20.9
	Very good		74	37.8
	Excellent		70	35.7
Information barriers	Yes		56	28.6
	No		140	71.4

Source: Field survey, 2020.

Multiple responses were also used to assess the types of information that respondents sought, such as the methods of infection and symptoms of COVID‐19, as well as the medicines used, health exercise, personal hygiene, treatment, isolation, and social distancing. About 75% of respondents sought information about the methods of infection, while 71% sought social distancing‐related information. About half of the respondents searched for isolation‐related information, while 41% sought information about COVID‐19‐related home treatment and personal hygiene as preventive measures. Only a few respondents showed interest in COVID‐19′s infection symptoms (6%) and health exercise (18%). In relation to overall information access, about 38% of respondents indicated that they had a very good experience, while 36% had an excellent experience and 21% had a good experience. Most respondents (71%) were not facing challenges in obtaining useful information.

### 4.3 Satisfaction with COVID‐19‐related information

Satisfaction with COVID‐19‐related information was assessed in terms of the amount, the quality, and the available sources of information. About 39% of respondents were highly satisfied with the amount of information, while 30% were satisfied and 25% were neutral. Similarly, most respondents (about 50%) were satisfied or highly satisfied with the information quality, while the remainder were either neutral or dissatisfied. Having available information sources was the key indicator of information satisfaction. Analysis showed that most respondents (about 51%) were satisfied with the availability of sources of information, while only 11% were dissatisfied (Table 4).

**TABLE 4:** COVID‐19‐related information satisfaction

	Extent of satisfaction (%)				
Variables	Very dissatisfied	Dissatisfied	Neutral	Satisfied	Highly satisfied
Satisfaction with the amount of information	3.1	3.6	24.5	29.6	39.3
Satisfaction with the quality of information	3.6	7.1	29.6	28.1	31.6
Satisfaction with the available sources of information	4.6	5.6	29.1	28.6	32.1

Source: Field survey, 2020.

#### 4.3.1 COVID‐19‐related information literacy

The level of information literacy on the COVID‐19 pandemic was assessed in terms of key indicators comprising: people's level of understanding; sharing COVID‐19‐related thoughts with others; credibility of the information; authenticity checking; decision making; and using information in daily life. The analysis showed that about 77% of respondents understood COVID‐19‐related information often or very often. About half of the respondents shared their COVID‐19‐related thoughts with others, while the remainder never, rarely, or sometimes shared these thoughts. Most respondents (72%) believed in the credibility of the information obtained, while the remainder believed in its credibility sometimes or rarely. Similarly, about 71% of respondents checked the authenticity of the information often or very often. Meanwhile, most respondents (68%) made decisions based on the collected information and used the information in their daily life (Table 5).

**TABLE 5:** COVID‐19‐related information literacy

	Extent of information literacy level				
Statements	Never	Rarely	Sometimes	Often	Very often
People's level of understanding	2	3.1	17.9	37.8	39.3
Sharing COVID‐19‐related thoughts with others	9.7	14.8	26	25	24.5
Credibility of COVID‐19‐related information	2	5.6	19.9	31.1	41.3
Checking authenticity of information	1.5	6.1	21.4	28.1	42.9
Making decisions based on information collected	3.6	4.6	20.4	33.7	37.8
Using information in daily life	3.1	2.6	23	33.2	38.3

Source: Field survey, 2020.

#### 4.3.2 COVID‐19‐related quality of life

Respondents were asked to give their opinions on their quality of life during the COVID‐19 pandemic. People worldwide faced numerous challenges in maintaining their quality of life owing to continuing lockdowns, social distancing, isolation, and restricted movement. In this situation, about 40% of respondents expressed the view that they had a lower quality of life during the COVID‐19 pandemic, while only 29% maintained their quality of life (Table 6).

**TABLE 6:** COVID‐19‐related quality of life

Statement	Major indicator	Frequency	Percentage
Do you think your quality of life has been normal during the COVID‐19 pandemic?	Yes	57	29.1
	No	79	40.3
	Maybe	60	30.6

Source: Field survey, 2020.

### 4.4 COVID‐19‐related depression, anxiety, and stress

The analysis showed that, during the COVID‐19 pandemic, about 42% of respondents felt tense always or most of the time in a day. Only 28.6% of respondents felt tense sometimes, whereas the remainder (about 30%) did not feel tense in relation to the COVID‐19 crisis. About 46% of respondents felt a slow‐down in their work or study very often or often, while 26.5% experienced this sometimes and 11.7% rarely. This study also compared the situation for respondents before and after the COVID‐19 pandemic began, revealing that, after the pandemic started, 33% of respondents enjoyed things a little or hardly at all, while 20% much enjoyed things and 25% moderately. Respondents also felt frightened owing to the COVID‐19 pandemic, rating this as ‘very often,’ ‘often,’ ‘occasionally,’ ‘rarely’, or ‘not at all.’ This study revealed that about 20% of respondents felt frightened very often or often, whereas others were frightened occasionally (26%), rarely (22%), or not at all (31%). Feeling frightened can create fear that awful events will happen, with respondents reporting that this occurred during the COVID‐19 pandemic, with a significant percentage of respondents facing this fear occasionally (23.5%) or rarely (24.5%). It is known that people also forget to take care of themselves during situations like the COVID‐19 pandemic. As the study's analysis found, about 16% of respondents indicated they had definitely lost interest in their appearance, while others reported this to different extents. People's ability to laugh at funny things was also rated compared to these feelings prior to the pandemic: the levels were feeling like they did before (47%), almost feeling like that (24%), and sometimes feeling like that (21.9%) (Table 7).

**TABLE 7:** COVID‐19 related depression, anxiety, and stress

Statements	Scales	Frequency	Percentage
Always feeling tense	Most of the time	39	19.9
	A lot of the time	43	21.9
	Sometimes	56	28.6
	Rarely	20	10.2
	Not at all	38	19.4
Feeling slow‐down in study/work	Very often	31	31.6
	Often	23	14.3
	Sometimes	52	26.5
	Rarely	23	11.7
	Not at all	31	15.8
Still enjoy things the same as before COVID‐19	Definitely as much	39	19.9
	Enjoy moderately	49	25
	Not quite so much	43	21.9
	Only a little	35	17.9
	Hardly at all	30	15.3
Always frightened	Very often	21	10.7
	Quite often	19	9.7
	Occasionally	51	26
	Rarely	44	22.4
	Not at all	61	31.1
Frightened feelings that something awful will happen	Very often	17	8.7
	Quite often	32	16.3
	Occasionally	46	23.5
	Rarely	48	24.5
	Not at all	53	27
Lost interest on appearance	I take just as much care as I did before	78	39.8
	I can't take care of my appearance properly	39	19.9
	I may not take quite as much care	33	16.8
	I don't take as much care as I should	15	7.7
	Definitely lost interest	31	15.8
Laughing at funny things	Definitely	92	46.9
	Almost	47	24
	Sometimes	43	21.9
	Rarely	11	5.6
	Not at all	3	1.5
Feeling restless	Very often	19	9.7
	Often	33	16.8
	Sometimes	48	24.5
	Rarely	47	24
	Not at all	49	25
Always thinking in my mind	Most of the time	19	9.7
	A lot of the time	27	13.8
	Sometimes	34	17.3
	Rarely	56	28.6
	Not at all	60	30.6
Looking forward to enjoying study	A great deal of the time	37	18.88
	A lot of the time	45	22.96
	From time to time, but not too often	81	41.33
	Only occasionally	33	16.84
Feeling cheerful with study	Most of the time	40	20.4
	A lot of the time	62	31.6
	Sometimes	77	39.3
	Rarely	12	6.1
	Not at all	5	2.6
Feeling sudden panic	Most of the time	11	5.6
	A lot of the time	15	7.7
	Sometimes	43	21.9
	Rarely	47	24
	Not at all	80	40.8
Relaxed during studying	Definitely	80	40.8
	Almost	49	25
	Sometimes	55	28.1
	Rarely	10	5.1
	Not at all	2	1
Enjoying the radio and TV programs	Very often	87	44.4
	Often	53	27
	Sometimes	38	19.4
	Rarely	12	6.1
	Not at all	6	3.1
Enjoying study/work	Often	31	15.82
	Sometimes	43	21.94
	Not often	79	40.31
	Very seldom	43	21.94

Source: Field survey, 2020.

Some people feel restless during periods of crises, such as pandemics. According to the current study's analysis, about 27% of respondents faced restless feelings while 7% experienced these feelings rarely or not at all. When experiencing stress, people feel like they always have something on their mind. The study's analysis showed that only 24% of respondents faced always having something on their mind, while some (17%) faced this sometimes, some (28.6%) rarely and some (30.6%) not at all. People tend to look forward to enjoying their work or study, but due to the COVID‐19 pandemic, 42% of respondents often could not look forward to enjoying work or study, while 17% only looked forward occasionally to enjoying work or study and the remainder looked forward to enjoying work or study sometimes. Most respondents felt cheerful sometimes during the COVID‐19 pandemic while others felt cheerful while studying or working. Most respondents (65%) did not experience feelings of sudden panic but others felt this to various levels of degree. About 66% of respondents felt relaxed while studying, while 28% felt relaxed sometimes. As a result of lockdowns and restricted movement, most respondents (44%) enjoyed the radio and TV programs, with only a few experiencing this enjoyment rarely. Most respondents (40%) experienced not enjoying their study or work often, with other respondents rating this as very seldom (22%), sometimes (22%), and often (16%).

### 4.5 Bivariate relationships between study variables

As can be seen in Table 8, age was positively correlated with access to ICT tools (*r*  =  0.23, *p* < 0.01), but negatively correlated with access to COVID‐19‐related information (*r* = −0.18, *p* < 0.05). Education level was negatively correlated with family size (*r *= −0.19, *p* < 0.05) and also with quality of life during the pandemic (*r* = −0.14, *p* < 0.05). The duration of staying in Sakaka city was negatively correlated with COVID‐19‐related information literacy (*r* = −0.19, *p* < 0.05). Family size was also negatively correlated with COVID‐19‐related information literacy (*r* = −0.17, *p* < 0.05) and with depression, anxiety, and stress (*r *= −0.15, *p* < 0.05). Access to COVID‐19‐related information had strong positive correlations with overall satisfaction (*r  *= 0.70, *p* < 0.01), and COVID‐19‐related information literacy (*r* = 0.52, *p* < 0.01). Moreover, satisfaction with COVID‐19‐related information was also positively correlated with COVID‐19‐related information literacy (*r* = 0.53, *p* < 0.01). COVID‐19‐related information literacy was also positively correlated with depression, anxiety, and stress (*r* = −0.15, *p* < 0.05).

**TABLE 8:** Correlation matrix of relationships between study variables

Variables	1	2	3	4	5	6	7	8	9	10
1 Age	1									
2 Edu	0.05	1								
3 Dur of stay	−0.01	−0.11	1							
4 Fam size	0.13	−0.19^*^	0.08	1						
5 Access to ICT tools	0.23^**^	0.08	0.02	0.09	1					
6 Access to COVID‐19 info	−0.18^*^	−0.09	−0.06	−0.05	−0.13	1				
7 Info sat	−0.08	−0.08	0.01	−0.03	−0.13	0.70^**^	1			
8 COVID‐19 lit	0.01	−0.08	−0.19^**^	−0.17^*^	−0.01	0.52^**^	0.53^**^	1		
9 QOL	0.04	−0.14^*^	0.01	−0.04	−0.12	0.08	0.09	0.06	1	
10 DAS	0.07	−0.03	−0.02	−0.15^*^	0.10	0.12	0.07	0.15^*^	−0.07	1

Note: ** Correlation is significant at 0.01 level; * correlation is significant at 0.05 level.

Edu, Education level; Dur of stay, Duration of stay; Fam Size, Family size; Info sat, Satisfaction with COVID‐19 information; COVID‐19 lit, COVID‐19 literacy; DAS, Depression, anxiety, and stress; QOL, Quality of life during COVID‐19 pandemic.

## 5 DISCUSSIONS

### 5.1 Advanced communication system (5G and 6G) for controlling COVID‐19

Wi‐Fi network services have extensively used by many hospitals to allow better connection, agility, positioning as well as localization communication [42]. Medical facilities, however, are tend to EMI (electromagnetic interference), that limits the application of Wi‐Fi networks services. Similarly, the heavy physical infrastructure of the hospital restricts the availability of high‐frequency wall signals and causes problems with network coverage. Sometimes, it is observed that Wi‐Fi network is inadequate to meet the need of communication in relations of capacity as well as connectivity during high patient loads [43]. In addition, real‐time health record monitoring via IoMT devices requires wide connectivity, high consistency and little latency. In the case of emergencies, a little delay and interrupted connectivity can cause serious consequences. In hospitals, largely reliable and minimum latency network is important, especially to allow robot technology to be used. It is also necessary to safeguard the network connection through advanced security systems to avoid unauthorized access to the confidentiality of patients and sensitive information [17].

Remote healthcare is being promoted with the emergence of COVID‐19 to lessen infection transmission and load of patient at the premises of hospital. Remote services are dependent on physical infrastructure for communication. Wireless networks are susceptible to safety threats like eavesdropping, blocking and spoofing, particularly in distant healthcare. If the communication network is not safe, the patient's critical information can be obtained by an eavesdropper or a spoofery. 6G Vision and Technologies Compared to earlier generations, 5G networks will offer better services with significant progress. The gaps in defense, intelligence, and wireless communication networking, that need the creation of advanced wireless networks should be ensures. Therefore, the vision of 6G is to use intelligence‐based communication tools to turn the world into a safe and interlinked digital society [25]. In the same manner as Symbolic Systems, Deep Learning does not process data. DL stores data in dispersed stores rather than systemic compositions of atomic units. In symbolic structures like Information Graphs, there's an interplay with Deep Learning. The arrangement of applications by input data type from a DL perspective can support readers to know the common issues. It avoids repeatedly explaining how a Deep Neural Network inputs language or images. Its programs that use the same type of input data have a lot in common. It is obvious that deep learning can be used to detect for COVID‐19 successfully.

### 5.2 Ensuring people's access to COVID‐19‐related information

The values produced in the analysis showed that most respondents were dependent on social networking sites for accessing information. It is well known that social networking sites not only possess authentic and useful information but also an enormous amount of fake and non‐scientific information. Fake information may cause serious health hazards. In the COVID‐19 pandemic period, social networking sites have been flooded with fake information that has caused numerous unexpected deaths worldwide. Therefore, public agencies could take the initiative to filter authentic information by using advanced digital technology. Respondents also tended to obtain information from health professionals, thus demonstrating good information‐seeking behaviour. However, a common problem in the study area and in other countries is that the number of health professionals is very much lower than the required number in terms of the population. Therefore, an alternative health information delivery system should be developed using advanced cloud technology to provide rapid information services to a huge population [19]. The study respondents showed a good level of acceptability of the ground rules, such as personal hygiene, social distancing, isolation, and mass testing that are controlling the rapid transmission of COVID‐19 in Sakaka city.

#### 5.2.1 Satisfaction with COVID‐19‐related information

People need and want readily available information to help to prepare for, and to protect themselves and recover from any infectious disease or virus. The study's findings showed that respondents were satisfied with information availability, quality, and sources. As satisfactory information empowers and motivates people, it can help them to fight a pandemic, such as COVID‐19. The government can ensure people's satisfaction with information access by delivering health‐related information rapidly and transparently using emerging digital technology.

#### 5.2.2 Ensuring COVID‐19‐related information literacy

Information literacy can enhance people's level of understanding, the credibility and authenticity of the information, as well as the ability to make decisions and implement measures in their daily life. The findings showed that respondents often understood the indicators of literacy but that a significant proportion had little interest in these issues. Therefore, various training programs, group meetings, leaflet distribution, and other context‐specific strategies should be undertaken by the government to improve people's health information literacy so they can protect themselves from the life‐threatening COVID‐19 infection.

#### 5.2.3 Ensuring COVID‐19‐related quality of life

Many people feel they are being deprived of their basic human rights owing to long‐term lockdowns, social distancing, restrictions on people's gatherings, and requirements to wear masks [4]. From a social perspective, people today have not experienced this kind of pandemic or its restrictions, as the previous one on this scale was over 100 years ago. Therefore, people are experienced boredom or fatigue with the need to maintain the ground rules of this health policy. They want to lead their lives as they did before the COVID‐19 pandemic but this would create more infection and greater loss of lives. These restrictions are hampering people's normal lives. Therefore, the government should take the initiative, relaxing the implementation of this health policy by ensuring technology‐based management of COVID‐19.

### 5.3 Solving depression, anxiety, and stress caused by COVID‐19

People's psychological health is being affected by the long‐term restriction of movement, lockdowns and social distancing, and forced vacations from the office and work. The numerous rules and stress about becoming infected with COVID‐19 are gradually creating psychological vulnerability, stress, anxiety, and depression as well as emotion [44]. The study's findings also showed respondents’ anxiety, depression, and vulnerability using a set of indicators. Ignoring these psychological issues will create long‐term mental health effects. Anxiety refers to fear of the unknown that develops due to continuous stress. Life‐threatening COVID‐19 transmission not only has an impact on the education level but also causes the disinterest in daily affairs that is known as depression. The rapid transmission of COVID‐19 has created significant levels of psychological vulnerability, anxiety, and depression among people worldwide. The unavailability of a vaccine, lack of effective medicines, and the rapidly changing nature of the coronavirus have created life‐threatening risks and vulnerability among all people. The long‐term pandemic has also created anxiety and depression among people, irrespective of their age [45, 46]. Therefore, timely steps should be taken to improve emotional health, with this being an urgent task.

In the study by Wang et al. [47] in which they explored psychological stress, many Chinese people were found to be facing psychological stress due to COVID‐19. Moreover, Qiu et al. [48] found that the level of psychological vulnerability was both alarming and common among Chinese people during the current COVID‐19 pandemic. Almost every infectious disease affects people's mental health but COVID‐19′s rapid spread and its effects are greater than for any other known disease or virus. As human beings, we, as individuals, are always interacting with others but this is now restricted by the requirement to maintain a social distance due to the coronavirus's nature of transmission to humans. Ease of access to information and technological support could reduce the transmission of COVID‐19, control its spread, and help to develop appropriate treatment.

### 5.4 Relationships between study variables

In exploring the bivariate relationships between study variables, age was positively correlated with access to ICT tools, with this being different to findings in other studies. It is generally assumed that people of a younger age have greater access to modern technologies and the use of ICT tools. There was a negative relationship between education level and quality of life during pandemic: the higher the level of education, the less the quality of life during the pandemic. It is generally believed that educated people have greater awareness and understanding of any unexpected situation and act accordingly. However, education level contributed to their anxiety which led to difficulties in their quality of life. This finding was in line with findings of many other researchers. Access to COVID‐19‐related information showed a strong positive correlation with overall satisfaction: This is easily understandable and corresponds with many other studies [8]. The study found that COVID‐19‐related information literacy was positively correlated with depression, anxiety, and stress. When people had more knowledge and literacy about the pandemic, they became more depressed, anxious, and stressed about the situation. This also corresponded with findings of some other studies.

## 6 CONCLUSION

Remote healthcare is being promoted with the emergence of COVID‐19 to lessen infection transmission and load of patient at the premises of hospital. Remote services are dependent on physical infrastructure for 4G and 5G communication. 5G Vision and Technologies Compared to earlier generations, 5G networks will offer better services with significant progress. Therefore, the vision of 5G is to use intelligence‐based communication tools to turn the world into a safe and interlinked digital society. This study attempts to focus on a combination of technological applications and people's experiences during COVID‐19 to formulate a suitable theoretical base for ensuring people's easy access to information systems (ISs). This study reveals several key areas in which technology could help to control the COVID‐19 pandemic through ensuring user‐friendly information systems (ISs). The study assesses the following five key aspects of the utilization of information by people during the pandemic: access to COVID‐19‐related information, COVID‐19‐related information satisfaction, COVID‐19‐related information literacy, COVID‐19‐related quality of life, and COVID‐19‐related depression, anxiety, and stress. This study reveals that most respondents receive information from social networking sites, health professionals, and TV without facing any challenges. The main types of information usually sought are for managing this health crisis, such as methods of infection, social distancing, and isolation. People also like to obtain useful information from available sources at a desirable quality. The level of COVID‐19‐related information literacy among respondents was not at the level expected which would require much attention from government agencies. People's overall quality of life was reduced due to this long‐term pandemic. The pandemic is also creating several psychological symptoms including anxiety, stress and depression, irrespective of people's age. For any safety and privacy threats, healthcare innovations anticipated in 5G should be vigorous technology. For example, during remote surgery, endwise linkage safety is essential for avoiding any undesirable interruption and attack from outsiders. As an artificial intelligence tool, deep learning (DL) can provide accurate results in helping to analyse lung diseases like COVID‐19 using CT images. Advancing from the robust learning capabilities of the feature, DL can automatically mine structures which are linked to clinical results. This study recommends that the government of every country initiate a national program though the integration of digital technology to ensure people's access to information to control the COVID‐19 pandemic. This study is based on only primary data from the respondents of a specific city, and similar kind of study can be extended in other places by increasing sample size and adding secondary data.
